# Despite Symptom Severity, do Nursing Home Residents Experience Joy-of-Life? The Associations Between Joy-of-Life and Symptom Severity in Norwegian Nursing Home Residents

**DOI:** 10.1177/08980101211021219

**Published:** 2021-07-02

**Authors:** Eva Rinnan, Beate André, Geir Arild Espnes, Jorun Drageset, Helge Garåsen, Gørill Haugan

**Affiliations:** 8018 NTNU Norwegian University of Science and Technology; 25574 Trondheim Municipality; NTNU Norwegian University of Science and Technology; 8018 NTNU Norwegian University of Science and Technology; 1657 Western Norway University of Applied Sciences; 1658 University of Bergen; Health and Welfare Services, Trondheim Municipality; 8018 NTNU Norwegian University of Science and Technology; 1786 Nord University

**Keywords:** nursing home, residents, joy-of-life, health-related quality of life, symptom severity

## Abstract

**Background:** Finding new approaches to increase health and well-being among nursing home (NH) residents is highly warranted. From a holistic perspective, several Norwegian municipalities have implemented the certification scheme framed “Joy-of-Life Nursing Home” **Aims:** In a holistic perspective on NH care, this study investigated if NH residents despite potential symptom severity experience joy-of-life (JoL). Therefore, we examined the frequency of common symptoms and the association between common symptoms and JoL in cognitively intact NH residents. **Methods:** A cross-sectional design was employed. Using the QLQ-C15–PAL quality-of-life questionnaire, hospital anxiety and depression scale, and JoL scale, a total of 188 cognitively intact NH residents participated. **Results:** Symptom severity was high; 54% reported fatigue, 52% reported constipation, 45% reported pain, 43% reported dyspnea, 32% reported insomnia, 22% reported appetite loss, and 20% reported nausea, while 20% reported anxiety and 23% reported depressive symptoms. Nevertheless, 59% of the NH residents reported high JoL, which was significantly positively related to the quality of life and negatively associated with anxiety and depression.

## Introduction

The 2030 Agenda for Sustainable Development states that a healthy life does not start or end at a specific age (United Natioan, 2015). By 2050, one in five people will be 60 years or older, totaling 2 billion people worldwide (United Nation, 2015). In Norway, the number of people aged 80 years or more will probably increase by 6–7% annually from 2025 to 2029, and the number of those who are 90+ will grow most rapidly (Ministry of Health and care services, H, 2014–2015). The extent of opportunities arising from increased longevity will depend on one key factor; the health status of these older populations (World Health Organization, 2018). The concept of healthy aging is defined as “the process of developing and maintaining the functional ability that enables wellbeing in older age” (World Health Organization, 2018)*.* Knowledge shows that well-being corresponds to holistic processes where people perceive a good life based on their own merits; well-being includes experiences such as joy, enjoyment, fulfillment, pleasure, satisfaction, happiness, relationships with family, and a sense of community (Söderbacka et al., 2017). Because of the increasing number of older adults living longer, the number of sick and frail elderly individuals in need of full-time care will rise (Ministry of Health and care services, H, 2014–2015). In 2018, ∼40,000 people were staying in Norwegian nursing homes (NHs) (Statistics Norway, 2020). Hence, knowledge about NH residents’ perceived joy-of-life (JoL) despite sickness, frailty, and symptom severity is strongly desired.

### Background

Generally, NH residents suffer from various losses, multiple, simultaneous, and complex illnesses, and diagnoses with severe symptom burden, impaired functioning, and fewer social relationships (Söderbacka et al., 2017). High incidence of chronic illness and functional impairments characterize long-term care residents, which require different types of medical treatment for palliation (Hoben et al., 2016). Typically, NH residents are characterized by frailty and vulnerability (Drageset et al., 2014; Tabali et al., 2013) and in Norway, ∼40% of all deaths each year are in NHs (Statistics Norway, 2020). Pain, dyspnea, incontinence, fatigue, and problems with personal hygiene are the most common physical symptoms in the NH population, while depression, anxiety, and loneliness are common psychological symptoms (Drageset, Eide, & Ranhoff, 2013; Beerens et al., 2013; Brownie & Horstmanshof, 2011; Erdal et al., 2017; Hoben et al., 2016). Therefore, in general, cognitively intact NH residents are in the last phase of life and in need of palliative care (Emilsdóttir & Gústafsdóttir, 2011). The quality of NH care will affect numerous individuals and their families worldwide. However, in general, nurses in long-term NH care are not sufficiently skilled in palliative care (Emilsdóttir & Gústafsdóttir, 2011). Consequently, inadequate pain management has been a significant challenge in NHs as well as alleviation of respiration symptoms (Campbell et al., 2018).

More frequently than older adults staying at home, NH residents suffer from depression and lack of social support. Symptoms of depression have been found unrecognized and inadequately treated in this population (Beerens et al., 2013; Emilsdóttir & Gústafsdóttir, 2011). NH residents report poorer health-related quality of life (QoL) than the general elderly population (Haugan, 2014b; Barca et al., 2009). In addition, the NH life is institutionalized, representing loss of social relationships, privacy, meaning in life, and connectedness (Haugan, 2014b; Barca et al., 2009). Insight into the prevalence of common symptoms such as pain, dyspnea, fatigue, insomnia, constipation, depression, and anxiety in NH residents is important with respect to QoL and health care quality. The QoL concept (Carlquist, 2015) has been used interdisciplinary with different understandings; from living conditions (objective) to feelings and emotions (subjective). This study utilized a holistic approach to well-being, using the concepts of QoL and well-being synonymously.

QoL and well-being represent important aims in NH care, which include more than treating residents` diseases and symptoms. Proper NH care includes a holistic approach to promote both mental and physical health and well-being. To enhance thriving for NH residents requires a shift from solely focusing on diseases and losses to a positive resource-oriented focus on creating experiences of health and a meaningful everyday life (Patomella et al., 2016). People living in NHs who experience thriving, have better functionality in activities of daily living, report higher QoL, and show less physical and cognitive impairments (Patomella et al., 2016). Both shared and individual activities are important for NH residents; living in a NH implies not only care and assistance, but also social needs; connecting and talking about one's life with somebody who is listening are significant (Gustavsson et al., 2015). Minor events and small changes might make a big difference in QoL for NH residents, especially if they have an active and meaningful role in developing their own activities (Boelsma et al., 2014). NH residents, who approach loss and hardships in life with optimism and hope, tend to experience higher levels of well-being (Brandburg et al., 2013).

To maintain dignity and continued personal growth in older adults, the 2030 goals for sustainable development (United Nations, 2015) underscores an urgent need for clinical research on the etiology and treatments of health conditions. Understanding the holistic processes that lead to health promotion for older adults is important. Accordingly, knowledge about the prevalence of common symptoms such as pain, dyspnea, fatigue, insomnia, constipation, depression, and anxiety in NH residents is important with respect to QoL, well-being, and care quality. Finding new approaches to holistically sustain NH residents’ health and well-being is highly warranted (United Nations, 2015).

### Joy-of-life Nursing Homes

The newly developed JoL concept (Rinnan et al., 2018; Haugan et al., 2019) seems closely related to subjective well-being, commonly defined as the absence of negative emotions, the presence of positive emotions, and life satisfaction (Carlquist, 2015), all of which corresponding to the concept of flourishing (Keyes, 2007; Seligman, 2010).

Rinnan et al. (2018) explored the phenomenon of JoL by interviewing 29 NH residents living in 10 Norwegian NHs revealing five dimensions: (1) positive relations, (2) belongingness, (3) sources of meaning, (4) moments of feeling well, and (5) acceptance (Rinnan et al., 2018). Based on theory, evidence, and these qualitative findings (Rinnan et al., 2018; Keyes, 2007; Seligman, 2010), a scale assessing perceived JoL in NH residents was developed and psychometrically tested (Haugan et al., 2019).

To promote JoL several Norwegian municipalities have implemented the certification scheme framed “JoL Nursing Homes” (JoLNH). The JoL Foundation developed and implemented the JoLNH strategy (Livsglede for eldre, 2021); based on a health promotion perspective the focus is on NH resident's resources, thriving, and well-being. Through health promotion, preventive and social activities across generations, the concept of JoLNH care promotes respect, well-being, health, relationships, meaningful activities, and cultural experiences among NH residents. To become a certified JoLNH, the individual NH must fulfill nine criteria concerning NH residents’ social, cultural, and spiritual needs. These nine JoLNH criteria have two purposes: (1) to facilitate experiences of JoL in NHs, which includes providing a meaningful everyday life and (2) to establish valid and appropriate documentation and evaluation routines and systems proving the fulfillment of these criteria. At present, 139 of about 942 Norwegian NHs have achieved the JoLNH certification or are in a process of certification (Livsglede for eldre, 2021; Statistics Norway, 2020).

### Aims

The aim of this study was twofold: (1) to investigate the frequency of common symptoms and (2) to explore the association between common symptoms and JoL in cognitively intact NH residents. The following hypotheses were tested:

**Hypothesis 1:** JoL is positively correlated with health-related QoL.
**Hypothesis 2:** JoL is negatively correlated with anxiety and depression.

## Methods

### Design

A cross-sectional design was employed using a questionnaire including the JoL Scale (JoLS), the European Organization for Treatment of Cancer (EORTC) quality of life questionnaire (QLQ-C15-PAL) which is a core palliative care questionnaire, and the hospital anxiety and depression scale (HADS). The 27 different NHs were invited one by one to participate until the minimum of *N*  =  200 participants were met. Out of 204 NH patients fulfilling the inclusion criteria, 188 participated with nearly no missing data giving a response rate of 92%. Listwise deletion resulted in *N*  =  181, which is the effective sample used in the statistical analyses. The inclusion criteria included that the NH resident (1) is capable of being interviewed and can express reflections and meanings, (2) has stayed in the NH for at least 3 months, and (3) is consent competent. NH residents with severe dementia and aphasia were excluded.

### Participants

The participants’ ages ranged from 63 to 104 years, with a mean of 87.4 years. The sample consisted of 133 women (73.3%) and 48 men (26.7%); the mean age was 88.3 years for women and 86 years for men. About 19.6% of the participants were married, 55.8% were widowers, and 24.3% were divorced or single (Table 1).

**Table 1.** Sample Characteristics

		Age (mean)	Residential time	Marital status
Total sample	*N* = 181	87.4 years	21 months (range: 3–124 months)	Married: 36 (19.9%)
				Widowers: 101 (55.8%)
				Divorced/single: 44 (24.3%)
Females	133 (73.3 %)	88.3 years		
Males	48 (26.7%)	86.0 years		

*Note*. *N* = 181.

### Ethics Approval and Consent to Participate

Individuals in NH’s represent a vulnerable population with difficulties in completing a large questionnaire. In this study, all participants were competent to give consent and voluntarily sign an informed consent form. Following a manual developed for this data collection, the interviewers were trained to conduct the interviews and taking notes after each interview in a similar way. The management in the participating municipalities, the leaders of the actual NHs, and the Regional Committee for Medical and Health Research in Mid-Norway approved this study.

### Data Collection

From March 2017 to May 2018, 204 individuals representing 27 NHs in two large cities and two small towns in Norway were included. The researchers contacted the management at the NHs and informed them about the study. A nurse in charge of the NH selected residents who fulfilled the inclusion criteria, provided them with oral and written information about the study, their rights as participants (to withdraw at any time), and collected a signed consent form. Then the researcher made an appointment with the NH residents to conduct the structured interview. The data were collected through individual interviews in the resident's private room. Six trained interviewers (including the first author) with an identical professional background as nurses conducted the interviews. The participants in this study have difficulties seeing, hearing, and writing due to their frailty, but they were able to reflect and answer the questionnaire. For clarity, the interviewers held a large print copy of questions and possible responses in front of the participant. The interviewers assisted the informants by reading every question and writing down the answers.

### Instruments

JoL was assessed by the JoLS, which was developed for this study in Norway (Rinnan et al., 2018). The intention was to identify essential characteristics of elderly individuals’ experiences of JoL in this life situation. Examples of JoLS items include feeling happy, having someone to love, close relationship with family and friends, experiencing meaning in life, and engaging in one’s surroundings (Appendix 1 in the supplemental material). The possible range of the JoLS is 13–91 for the validated 13-item version (Haugan et al., 2019), which was used in this study.

*Common symptoms* were assessed by EORTC QLQ-C15-PAL, which is a core palliative care questionnaire (Groenvold et al., 2006) assessing common symptoms such as pain, fatigue, etc. The QOL-C15-PAL is an abbreviated 15-item version of the EORTC QLQ-C30, made up of two multi-item functional scales (physical and emotional functioning), two multi-item symptom scales (fatigue and pain), five single-item symptom scales (nausea/vomiting, dyspnea, insomnia, appetite loss, and constipation), and one final question referring to overall QoL. Each item is rated on a numeric scale from 1 (not at all) to 4 (very much), except for the global QoL, which is rated from 1 (very poor) to 7 (excellent). The QLQ-C15-PAL has demonstrated good content validity (Groenvold et al., 2006). This study used the Norwegian version validated for cancer patients (Groenvold et al., 2006).

*Anxiety and depression* were assessed by the HADS, comprising 14 items with subscales for anxiety (HADS-A; seven items) and depression (HADS-D; seven items). Each item is rated from 0 to 3, where higher scores indicate more anxiety and depression. The maximum score is 21 on each subscale. The HADS has been extensively tested showing well-established psychometric properties (Herrmann, 1997; Norton et al., 2013). It has been translated into Norwegian and found valid among older people (Stordal et al., 2001; Stordal, Mykletun, & Dahl, 2003) as well as validated among cognitively intact NH residents, showing good to acceptable reliability and validity (Haugan & Drageset, 2014).

### Statistical Analysis

Statistical analyses were performed using STATA version 15.1 (StataCorp, 2017). Descriptive statistics were performed to describe the level of JoL, QoL, and HADS. Correlation analyses were performed to determine the relationship between JoL, QoL, and anxiety and depression. The hypotheses were tested using correlation analyses and measured by Pearson’s *r*^2^. Missing values were deleted listwise.

## Results

**Figure 1.**
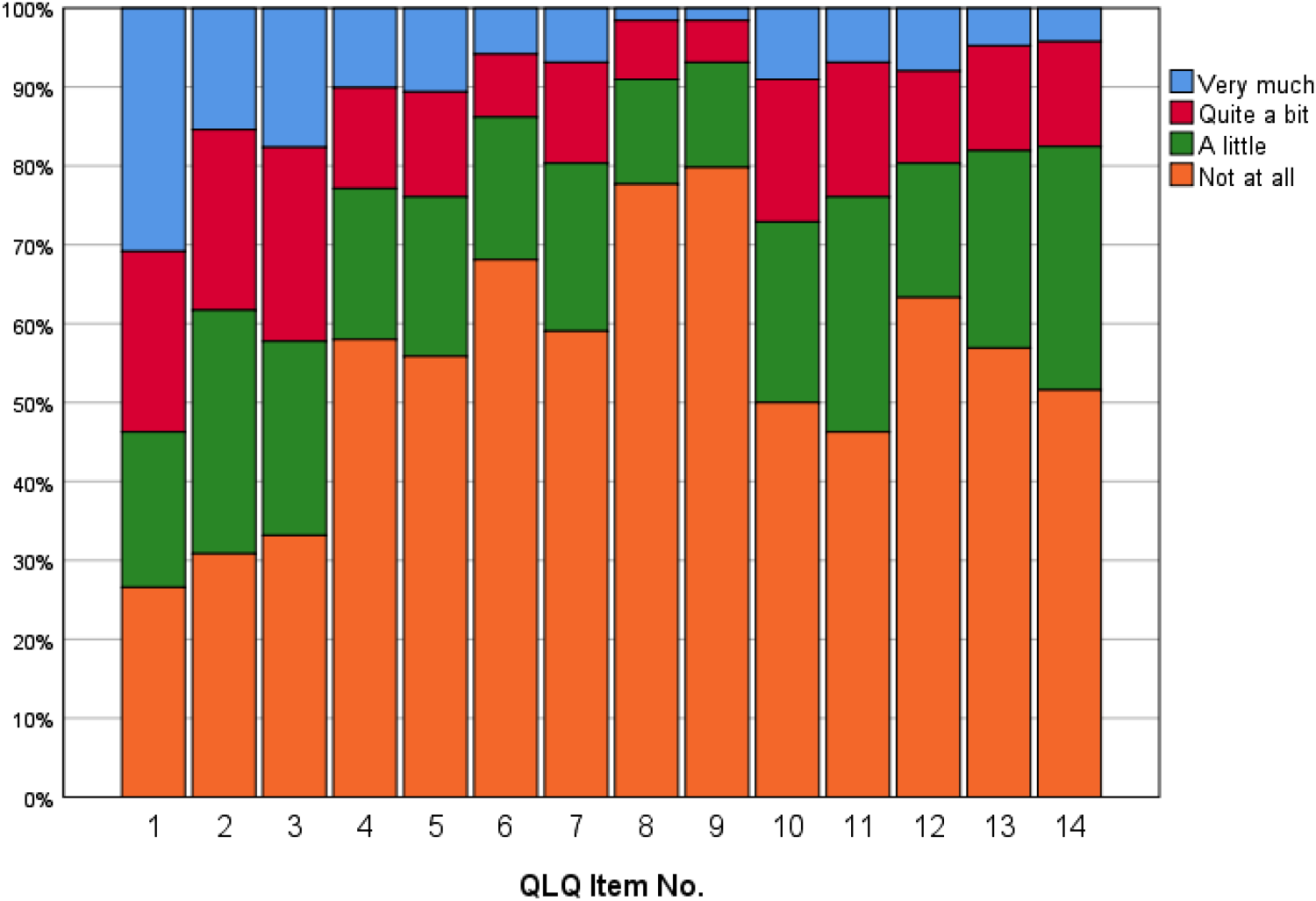
The prevalence of symptoms among nursing home residents. *N* = 181.

*Note.* The prevalence of physical and emotional symptoms assessed using the quality of life questionnaire core 15 palliative care (QLQ-C15-PAL) items 1–14. Item 1: …difficulties with walking outside. Item 2: …necessary to rest in bed or sit in a chair. Item 3: … need help to dress, wash or go to the toilet. During the past week… Item 4: …did you have difficulties with breathing. Item 5: …did you have pain. Item 6: …did you have problems with sleeping. Item 7: …did you feel week. Item 8: …did you have bad appetite. Item 9: …did you experience nausea. Item 10: …did you experience constipation. Item 11: …did you feel tired. Item 12: …have pain affected your daily activities. Item 13: …did you feel tense. Item 14 …did you feel depressed.

Figure 1 shows the prevalence of symptoms, based on the frequency of the QLQ-C15-PAL items. The figure portrays the NH residents self reported symptom severity sorted by; not symptoms at all (orange), a little (green), quite a bit (red), and very much symptoms (blue). The α-levels for the various measures indicated an acceptable level of inter-item consistency with a Cronbach's alpha coefficient of 0.88 for the JoL 13-item scale, 0.84 for the QLQ-C15-PAL 14 items, and 0.83 for the HADS total scale.

Figure 1 shows that about 70% of the residents reported severely reduced physical functioning involving difficulties in walking, spending much time in bed, and the need for help in washing and toileting (QLQ physical functioning). Fatigue (54%), constipation (52%), pain (45%), dyspnea (43%), and insomnia (32%) were the most common physical ailments, while 22% reported appetite loss and 20% had nausea. Concerning emotional functioning, about 45% reported anxiety and nearly 50% reported depression. However, looking at the HADS scores, 20% had anxiety whereas 23% reported depressive symptoms; among these stated 14% mild depression, 6% moderate, and 3% severe depression (Table 2).

**Table 2.** Anxiety and Depression. The Distribution of the Hospital Anxiety and Depression Scale (HADS) Scores

HADS score	HADS_anxiety (%)	HADS_depression (%)
Normal	80	77
Mild disorder	10	14
Moderate disorder	8	6
Severe disorder	2	3

*Note.* HADS scores are interpreted as follows: Normal = score 0–7, mild disorder = score 8–10, moderate disorder = score 11–14, and severe disorder = score 15–21.

Evaluating JoL, the analyses showed a mean of 4.78 (*SD* 1.28). The cut-off values are not statistically defined but interpreted by common sense; scores between 5 and 7 were interpreted as a high JoL, while scores between 4.0–4.9 and 1–3.9 were interpreted as indecisive and low JoL, respectively. In this study, 59% (*N*  =  107) of the NH residents reported high JoL (≥5.0), 15.5% (*N*  =  44) reported indecisive JoL (4.0–4.99) and 25.65% (*N*  =  46) reported low JoL (0–3.99) (Table 3). More specific, about 91.5% reported that during the last week contact with their family and friends made them happy, 69.2% had contact with the world outside, 68.6% accepted themselves as they are, 67.4% had someone to speak with in confidence, 66.5% were grateful for how life had become, and 63.8% had experienced something that made them happy. The lowest scores were displayed for perceived meaning in everyday life (35.6%), feeling valuable (35.8%), engaging in the surroundings (36.7%), a sense that one contributes to others (42.6%), and having something meaningful to fill the days with (43.6%).

**Table 3.** The Distribution of Joy-of-Life Among the Nursing Home Residents (Joy-of-Life Scale [JoLS], 13 Items)

During the last week, to what extent have you experienced that you…	Score: 1–3 low (%)	Score: 4 medium (%)	Score: 5–7 high ( %)
JoLS1…*feel happy during the day in the nursing home*	17.0	25.0	58.0
JoLS2…*experience meaning in your everyday life*	35.6	19.7	44.6
JoLS3… *have a good balance between activity and rest*	18.5	28.5	52.7
JoLS4…*engage in your surroundings*	36.7	11.7	51.5
JoLS5…*experience something that makes you happy*	15.4	20.7	63.8
JoLS6…*contact with your family makes you happy*	5.3	3.2	91.5
JoLS7…*feel valuable*	35.8	14.4	49.7
JoLS8…*have something meaningful to fill your days with*	43.6	17.0	39.4
JoLS9…*feel that you can contribute positively to others*	42.6	13.3	44.2
JoLS10…*have someone to speak with in confidence*	23.5	9.1	67.4
JoLS11…*feel grateful for how your life is*	19.2	14.4	66.5
JoLS12…*accept yourself as the person you now are*	18.1	13.3	68.6
JoLS13…*are in contact with the world outside the nursing home*	19.7	11.2	69.2

*Note. N* = 181.

Table 4 displays Cronbach's alpha and the Pearson's correlation matrix for the study variables while Appendix 1 in the Supplemental Material describes the distribution of the JoL scores, means (*M*), and *SD*.

**Table 4.** Correlation Coefficients and Cronbach’s Alpha for the Study Variables

Variables	Pearson’s *r*^2^ JoL mean 13 items
JoL mean (13 items)	1.00
QLQ PF2	0.16*
QLQ FA2	−0.18*
QLQ NV	−0.06
QLQ EF	0.39**
QLQ PA2	−0.14
QLQ DY	−0.18*
QLQ SL	−0.23**
QLQ AP	0.06
QLQ CO	−0.20**
QLQ total	0.47**
HADS Anxiety	−0.39**
HADS Depression	−0.65**
HADS total	−0.60**
Cronbach’s alpha	
JoL 13 items	0.88
QLQ 14 items	0.84
HADS 14 items	0.83

*Note*. *N* = 181. JoL = joy-of-life; PF2 = physical functioning; FA2 = fatigue; NV = nausea and vomiting; EF = emotional functioning; PA2 = pain; DY = dyspnea; SL = insomnia; AP = appetite loss; CO = constipation; QLQ = quality of life questionnaire; HADS = hospital anxiety and depression scale.

*Significant at the 5% level. **Significant at the 1% level.

### Relationships between JoL and Common Symptoms

The correlations between JoL and symptom severity (Table 4) were moderate. Pearson's correlation coefficient (*r*^2^) displayed significant values for the JoL construct to all symptoms, except pain, appetite loss, nausea, and vomiting. The highest correlations were found between JoL and depression (−0.65), overall QoL (0.47), anxiety (−0.39), and emotional functioning (0.38). Although moderate values, significant correlations were revealed between JoL and physical functioning (0.16), insomnia (−0.23), fatigue (−0.18), constipation (−0.20), and dyspnea (−0.17).

**Figure 2.**
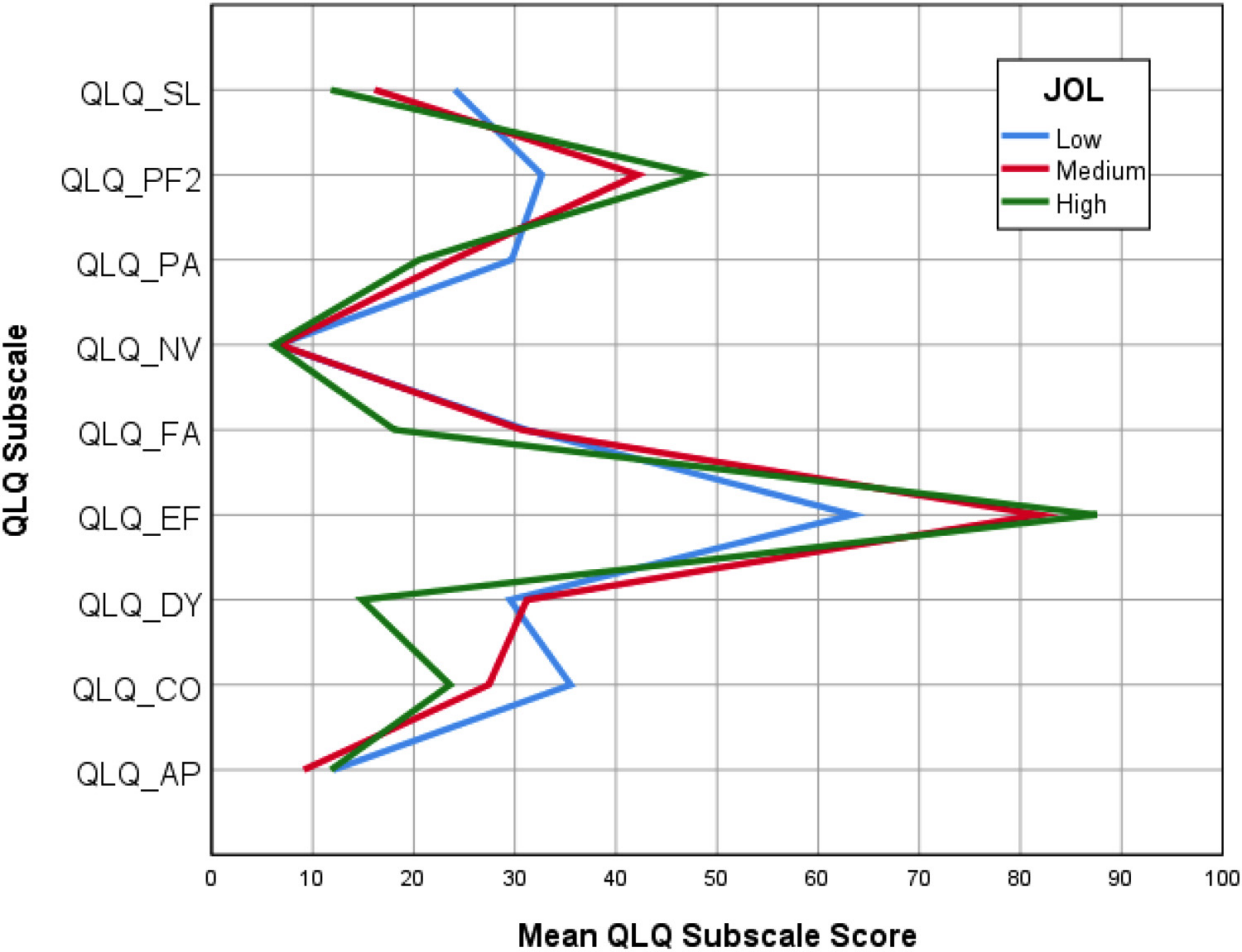
Symptom severity associated with JoL sorted by high, medium, and low JoL.

*Note*. QLQ versus JoL. The symptom profiles for groups of NH patients based on high, medium or low JoL score: JoL scores are based on the 33.33 and 66.67 percentiles; JoL ≤ 33.33 = low JoL, JoL ≥ 33.33 and ≤66.67 = medium JoL, and JoL ≥ 66.67 = high JoL. Symptoms are assessed by means of the QLQ-C15-PAL subscales: QLQ_SL, QLQ_PF2, QLQ_PA, QLQ_NV, QLQ_FA, QLQ_EF, QLQ_DY, QLQ_CO, and QLQ_AP. JoL = joy-of-life; NH = nursing home; QLQ = quality of life questionnaire; QLQ-C15-PAL = quality of life questionnaire core 15 palliative care; SL = insomnia; PF2 = physical functioning; PA = pain; NV = nausea and vomiting; FA = fatigue; EF = emotional functioning; DY = dyspnea; CO = constipation; AP = appetite loss.

Figure 2 shows the association between JoL and symptom severity: JoL is sorted by high (green line), medium (red line), and low (blue line). Figure 2 illustrates that high and medium JoL are associated with lower symptom severity and thus better health-related QoL. The JoL scores were based on the 33.33 and 66.67 percentiles: JoL ≤ 33.33  =  low JoL, JoL ≥ 33.33 and ≤66.67  =  medium JoL, and JoL ≥ 66.67  =  high JoL. As shown in Figure 2, patients reporting a high and medium JoL showed better physical and emotional function and less symptom burden than residents reporting lower JoL, revealing more severe symptoms (except for insomnia, pain, and constipation) and lower physical and emotional functioning (Hypothesis 1).

## Discussion

Holistic knowledge about NH residents perceived JoL despite sickness, frailty, and symptom severity is strongly desired. Therefore, the aim of this study was to investigate symptom severity and the association between common symptoms and JoL among cognitively intact NH residents. By doing so, this study provides novel knowledge about NH residents’ experience of JoL and symptom burden in three ways: (1) portraying the prevalence of common symptoms in the NH population; (2) providing evidence on the association between common symptoms and the experience of JoL; and (3) presenting insights about the associations between symptom severity and JoL among NH residents.

Mean age of the participants were 88.3 years for woman and 86 years for men, which is in accordance with previous NH studies (Haugan, 2014b; Halvorsen et al., 2017). JoL was strongly and positively correlated with total QoL (Hypothesis 1), but not with pain, appetite loss, and nausea. Therefore, Hypothesis 1 found partly support. Furthermore, JoL was strongly negatively correlated with depression and anxiety (Hypothesis 2), and accordingly emotional functioning. Hence, Hypothesis 2 found support.

The results showed that about 70% of the NH residents reported severely reduced physical functioning involving difficulties in walking, spending much time in bed, and the need for help in washing and toileting (QLQ physical functioning). Symptom severity was high, revealing that 54% reported fatigue, 52% reported constipation, 45% reported pain, 43% reported dyspnea, and 32% reported insomnia, while 22% reported appetite loss and 20% reported nausea. These figures correspond with two previous studies among NH residents showing a similar symptom severity (Haugan, 2014b; Hoben et al., 2016). Accordingly, it is plausible that symptom severity in this population is high. Despite the high ages, several simultaneous diagnoses (Fortin et al., 2012; Marengoni et al., 2011), residential time about 1–2 years (Hjaltadóttir et al., 2011; Vossius et al., 2018), and that 40% of deaths annually occurs in Norwegian NHs (Statistics Norway, 2020), still focus on palliation is scarce in NHs (Emilsdóttir & Gústafsdóttir, 2011).

Moreover, the NH residents displayed low emotional functioning; 45% reported anxiety, and close to 50% reported depressive symptoms. Furthermore, the HADS scores indicated 20% anxiety and 23% having symptoms of mild (14%), moderate (6%), and severe (3%) depression. These findings are in accordance with previous studies, showing increased prevalence of depression in old age (Haugan, 2014b; Barca et al., 2010; Solhaug et al., 2012), demonstrating that various losses often lead to geriatric depression, and that physical illness and disability represent strong influences in the development and persistence of depression (Barca et al., 2010). However, previous research has highlighted that symptoms of depression often are overlooked and untreated in NHs (Iden et al., 2014; Prado-Jean et al., 2011). Simultaneously with the reports of high depressive burden are the reports of NH residents struggling with existential questions. Existential issues can be described as fundamental issues of human life such as what makes life worth living and how do I cope with the finality of my life. Difficulties to find answers to these questions can result in existential suffering and distress (Grech & Marks, 2017). The few studies available show the need of NH residents to talk about existential issues (Haugan, 2014c, 2014d, 2014e; Haugan et al., 2013; Sjöberg et al., 2018; Smedbäck et al., 2017). Lately, perception of existential suffering has become the focus of research in cancer care and palliative medicine (Gautam et al., 2019; Kissane, 2012), but is hardly studied in the NH context. Still, NH residents are daily confronted with losses, disease, a severe symptom burden, and bereavement, as well as time spent in passive activities, such as doing nothing, sleeping, and waiting, which lead to feelings of boredom, loneliness, meaninglessness, and indignity (Brownie & Horstmanshof, 2011; Slettebø et al., 2017). Consequently, existential issues such as life's finality, social isolation, and meaninglessness seem actual in the NH population and may shed light on the present scores on emotional functioning assessed by the QLQ-C15-PAL scale diverging from the HADS scores assessing anxiety and depression.

The present results show that despite the high symptom burden with low physical and emotional functioning, JoL was quite high (mean score 4.8, range 1–7). Looking closer into the results, this study shows that contact with family, friends, and the world outside the NH contributed to JoL and happiness. These results indicate that the need for belonging; that is, having someone to love and at the same time be loved and cared for, is important for JoL in NH residents (Drageset et al., 2008; Rinnan et al., 2018; James et al., 2014) as well as signifying that people might flourish in late life (Gautam et al., 2019) if given the right opportunities. Tilvis et al. (2012) found that feeling needed represents a dimension close to love and being loved, and is an important predictor of a survival prognosis in old age (Tilvis et al., 2012). Moreover, Snowden et al., (2018) found that being able to talk to somebody about what is on your mind is crucial and the only necessary in spiritual care. Hence, talking to nurses, also termed nurse–patient interaction (Haugan, 2021), and others about what is on one’s mind seem essential to well-being among NH residents (Haugan et al., 2020). Professionals in NHs should facilitate possibilities for NH residents to have visitors and contact with friends and family.

In this study, despite a heavy symptom burden, about 60% experienced something in their daily life making them happy. This may indicate that they had some activities and social contacts during the day which were enjoyable. About 70% felt grateful for how their life was and accepted the person they had become. According to Meeks and Looney (2011), the ability to regulate positive affect to maintain more positive than negative emotions is an important aspect of successful adjustment in late life (Meeks & Looney, 2011).

Also, about 70% shared that they had someone to speak with in confidence, indicating that they were not lonely, as well as had someone to talk to about what was on their mind (Snowden et al., 2018). A differentiation of the phenomenon “loneliness” among NH residents might be useful; Sundström et al. (2018) described existential loneliness as related to feeling disconnected from the world, lost without a purpose, and a drift in life. Existential loneliness can also arise when people lack previous experiences relevant to their present situation or in times of uncertainty such as during an illness (Sundström et al., 2019). This description might be suitable to the experience of old people living in NHs, but at the same time, NH residents also need to spend time alone in silence talking to themselves and resting their minds (Drageset et al., 2017; Haugan, 2014a, 2021). The present results disclosed the lowest scores for perceived meaning in life, feeling valuable, and being able to contribute to others, as well as engaging in one's surroundings and meaningful activities. Slettebø et al. (2017) concluded that meaningful activities are important for NH residents to experience dignity and underscored that the activities should be in preference and planned together with the resident (Slettebø et al., 2017).

A lack of meaning in life might indicate an existential demanding life situation, in which feeling valuable and contributing to others prove difficult. Meaning in life is found essential to NH residents’ well-being, both emotionally, socially, spiritually, and physically (Haugan, 2014c; Haugan et al., 2020), and life satisfaction (Haugan, 2014a; Prieto-Flores et al., 2011). Therefore, attention toward these qualities in an old person's life should be included in NH care, assessing NH resident's experience of value, meaning, and life satisfaction.

Furthermore, this study showed that JoL correlated significantly with almost all symptoms. However, we found no significance with pain, appetite loss, nausea, and vomiting. One might expect that perceived JoL would be negatively and significantly associated with pain, appetite loss, nausea, and vomiting: especially pain is found to significantly impact people's QoL (Campbell et al., 2018; Emilsdóttir & Gústafsdóttir, 2011). The present results indicate that JoL was not affected by pain, appetite loss, and nausea, which is surprising. Hence, this correlation needs further elaboration. Nevertheless, in light of the reported symptom severity, pain and symptom management are highly needed in the NH population: a recent study showed that perceived loneliness among NH residents was significantly influenced by the fact that the nurses made all possible effort to relieve the NH residents’ ailments (Drageset & Haugan, 2021). Consequently, holistic palliative care should be an integrated part of good NH care.

### Limitations and Strengths

In total, 204 NH residents fulfilled the inclusion criteria, among them 188 residents participated, giving a response rate of 92% representing strength of this study. The questionnaire comprised 132 items, thus frail older NH residents might tire when completing the questionnaire. This might represent a possible bias to their reporting. To minimize such a bias, we carefully selected and trained experienced researchers in conducting the interviews following the exact same procedure, taking small breaks at specific points during the interviews. All participants fulfilled the questionnaires without considerable difficulties. The fact that the researchers visited the participants to help fill in the questionnaire might have introduced some bias on the respondents’ responses, which might be considered a strength concerning accurateness, but also a limitation when it comes to possible unintended influences.

## Conclusion

This study shows that symptom severity is high among NH residents. JoL was shown to be strongly positively associated with several aspects of health-related QoL, and strongly negatively associated with anxiety and depression.

Despite low physical and emotional functioning and a high symptom burden, we found that largely, NH residents reported to be happy, experienced enjoyable social activities, and positive contact with friends and family, and were grateful for how their life had become, and accepted the person they had been in life. This indicates that NH residents accept and adapt to their life situation as well as adjusting their orientation to life in their late years.

Still, improvements in NH care are needed; pain and symptom management should be prioritized as well as a nurse–patient interaction (Haugan, 2014d; Haugan et al., 2013; Haugan et al., 2020). Also, efforts to facilitate NH residents’ perceived meaning in life, making them feel valuable, while providing opportunities for them to contribute to other people's lives, still being involved in others, the surroundings, and meaningful activities, are crucial for JoL in this population.

## Author Contribution

All authors fulfilled at least one of the following criteria recommended by the medical journal editors (ICMJE):

- Substantial contributions to conception and design, acquisition of data, or analysis and interpretation of data.
- Drafting the article or revising it critically for important intellectual content.

## Supplemental Material

sj-doc-1-jhn-10.1177_08980101211021219 - Supplemental material for Despite Symptom Severity, do Nursing Home Residents Experience Joy-of-Life? The Associations Between Joy-of-Life and Symptom Severity in Norwegian Nursing Home Residents Click here for additional data file. Supplemental material, sj-doc-1-jhn-10.1177_08980101211021219 for Despite Symptom Severity, do Nursing Home Residents Experience Joy-of-Life? The Associations Between Joy-of-Life and Symptom Severity in Norwegian Nursing Home Residents by Eva Rinnan, Beate André, Geir Arild Espnes, Jorun Drageset, Helge Garåsen and Gørill Haugan in Journal of Holistic Nursing
