# Supplementary material for: Despite Symptom Severity, do Nursing Home Residents Experience Joy-of-Life? The Associations Between Joy-of-Life and Symptom Severity in Norwegian Nursing Home Residents
Source: J Holist Nurs. 2021 Jul 2;40(2):84–96. doi: 10.1177/08980101211021219 (PMC9121523; doi:10.1177/08980101211021219)
Supplement: sj-doc-1-jhn-10.1177_08980101211021219 - Supplemental material for Despite Symptom Severity, do Nursing Home Residents Experience Joy-of-Life? The Associations Between Joy-of-Life and Symptom Severity in Norwegian Nursing Home Residents [file sj-doc-1-jhn-10.1177_08980101211021219.doc]

**APPENDIX 1** The level of JoL among 181 Nursing home residents

**The Joy-of-Life Scale (JoLS).** The 13-items version. Means and Standard deviation

*During the last week, to what extent have you experienced that you…*

| **Variable** | **Mean** | **Std.Dev.** |
| --- | --- | --- |
| JoLS1…*feel happy during the day at the nursing home* | 4.792553 | 1.606986 |
| JoLS2…*experience meaning in your everyday life* | 4.095745 | 1.887585 |
| JoLS3 *have a good balance between activity and rest* | 4.698925 | 1.732269 |
| JoLS4…*engage in your surroundings* | 4.31383 | 2.094664 |
| JoLS5…*experience something that makes you happy* | 5.015957 | 1.756504 |
| JoLS6…*contact with your family makes you happy* | 6.335106 | 1.274783 |
| JoLS7…*feel valuable* | 4.336898 | 2.201875 |
| JoLS8…*have something meaningful to fill your days with* | 3.781915 | 1.909809 |
| JoLS9…*feel that you can contribute positively to others* | 3.898936 | 2.087754 |
| JoLS10…*have someone to speak with in confidence* | 5.069519 | 2.102349 |
| JoLS11…*feel grateful for how your life is* | 5.085106 | 2.016825 |
| JoLS12…*accept yourself as the person you now are (or have become)* | 5.212766 | 1.906209 |
| JoLS13…*are in contact with the world outside the nursing home* | 5.255319 | 1.961862 |
| Mean : 13 items Joy of Life | 4.776 | 1.232 |
